# Physical Activity Programming for Older Adults in Assisted Living: Contextual Factors to Consider

**DOI:** 10.1093/geroni/igab046.3724

**Published:** 2021-12-17

**Authors:** Katelyn Webster, Janet Larson

**Affiliations:** University of Michigan School of Nursing, Ann Arbor, Michigan, United States

## Abstract

Sedentary behavior may adversely affect physical and cognitive health of older adults in assisted living (AL). Replacing sedentary behavior with light physical activity (PA) could help them maintain functional abilities and independence. We interviewed AL residents to obtain their guidance regarding the implementation of an intervention to reduce sedentary behavior. Here we report the results of a thematic analysis exploring contextual factors that may influence intervention implementation. We interviewed 20 residents (mean age 83.1; 60% women) and identified 7 themes. The first was attitudes and beliefs about PA. Most residents believed PA was important, but some lacked motivation or confidence to perform PA. Another theme was attitudes and beliefs about aging, as some residents felt discouraged about aging and uncertainty about how much PA they could safely perform. Abilities of AL residents was seen as an important consideration. It was noted that residents have a wide range of abilities and this could present challenges in planning a PA program appropriate for all residents. Social influences for PA should be considered, as residents may find encouragement from family or other residents. Space for being active is another factor because it is typically limited within AL. We found that some residents wanted more challenging exercise classes than currently provided by their facility. Finally, residents described limited opportunities for PA due to the nature of the AL environment. This thematic analysis brings attention to important factors that could influence the implementation of PA interventions with the AL population.

